# Supplementary material for: Relating parental stress with sleep disorders in parents and children
Source: PLoS One. 2023 Jan 25;18(1):e0279476. doi: 10.1371/journal.pone.0279476 (PMC9876271; doi:10.1371/journal.pone.0279476)
Supplement: Attachment — Submitted filename: Response to Reviewers.docx [file pone.0279476.s002.docx]

Response to Reviewer Comments:

I would think twice about the word “According” in the title. It directs readers in a different path.

Response: The title has been changed to now say: “Relating Severe Stress with Sleep Disorders in Parents and Children”

- The objective is unclear

Response: The objective in the Abstract now says: “To assess whether child sleep disorder positively correlates with parental insomnia, hypersomnia, and sleep apnea, and to identify and whether parental and child sleep disorders simultaneously positively associate with parental stress. Potential modifying influences of these associations by age, sex, and marital status will be considered.” This objective is consistent with what is written at the end of the Introduction, in the Discussion, and in the Conclusion.

- In the abstract there are 2 sentences that say the same thing –
The rate of stress is 2.13 (95% CI 1.62–2.78) times higher in those with a sleep disorder after adjusting for age, sex, and marital status.

Response: The Abstract has been rewritten to better align with the clarified objective and hypotheses of the study. These sentences are no longer included.

Among contract holders, mothers, singles, and those with a sleep disorder had significantly higher rates of stress
If that is not so – then a clarification is needed.

Response: This statement is consistent with the findings.

- I suggest using the term “participants” instead of “contract holders”. This does not feel as an academic terminology.

Response: Because all enrollees (parents and children) in the study are participants, and because we wanted to distinguish between the contract holders and the children, we thought the word “employees” would work better for “contract holders.”

- Conclusions -not innovative.

Response: the Conclusion section in the Abstract and at the end of the paper tries to emphasize the importance of this study.

- Last sentence p2 – but also a possible predictor of forthcoming mental health disorders - why is this relevant? Needs clarifying.

Response: The sentence was deleted.

- Hypotheses are needed.

Response: Four specific hypotheses were added at the end of the Introduction. The Discussion is organized by these four hypotheses.

- The introduction explains previous research on the given subject but does not show how this study adds on previous knowledge.

Response: The last paragraph of the Introduction has been rewritten and clarifies what this study adds.

- Population –
o Data given is lacking. I don’t need to hear about the insurance company but data about the participants that may be relevant to stress and parenting. Such as: gender prevalence, mean age, socioeconomic status, marital status, family orientation, hours of work outside the house, etc.

Response: Under the section titled Data Collection in the Methods, a little more information about the study group is added (i.e., the distribution of salaries for the employees). The first paragraph of the Methods includes a description of the enrollees in DMA according to place of residence and job type.

- Discussion
This study identified the rate of parental stress and sleep disorders and the association between these variables. That is interesting but not adding to existing knowledge.

Response: The Introduction (last paragraph) now clarifies what is known and what will be added. The Discussion begins by addressing how the Results provide information consistent with the literature. Discussion about the hypotheses, however, provides information on what knowledge is being added by this study.

o Suggestions for future research are needed.

Response: In the Discussion we add that “The null result involving hypersomnia is inconsistent with previous research [32, 33] and deserves further investigation.” Also, a limitation paragraph was added at the end of the Discussion. One sentence included there says: “However, future research may link additional years of data in order to better understand temporal sequences of events between sleep disorders and stress.”

o What are the practical implications of this research?

Response: The following was added at the end of the Conclusion: “Better understanding the connection between parent and child sleep quality and parent stress, and modifying influences, may help improve treatment and lower the risk of these disorders.”
